# cGAS regulates the DNA damage response to maintain proliferative signaling in gastric cancer cells

**DOI:** 10.32604/or.2022.03529

**Published:** 2022-07-13

**Authors:** BIN LIU, HAIPENG LIU, FEIFEI REN, HANGFAN LIU, IHTISHAM BUKHARI, YUMING FU, WANQING WU, MINGHAI ZHAO, SHAOGONG ZHU, HUI MO, FAZHAN LI, MICHAEL B. ZHENG, YOUCAI TANG, PENGYUAN ZHENG, YANG MI

**Affiliations:** 1 Henan Key Laboratory of Helicobacter Pylori & Microbiota and Gastrointestinal Cancer, The Fifth Affiliated Hospital of Zhengzhou University, Zhengzhou, China; 2 Academy of Medical Science, Zhengzhou University, Zhengzhou, China; 3 Clinical and Translational Research Center, Shanghai Pulmonary Hospital, Tongji University School of Medicine, Shanghai, China; 4 Gastrointestinal Surgery, The Fifth Affiliated Hospital of Zhengzhou University, Zhengzhou, China; 5 Department of Gastrointestinal Surgery, Henan Cancer Hospital, The Affiliated Cancer Hospital of Zhengzhou University, Zhengzhou, China; 6 Gastrointestinal Surgery, People Hospital of Zhengzhou University, Zhengzhou, China; 7 Faculty of Science, School of Interdisciplinary Science, Honours Life Sciences, McMaster University, Hamilton, ON, Canada

**Keywords:** Gastric cancer, Cell proliferation, cGAS, DNA damage response, MRN complex

## Abstract

The activation of some oncogenes promote cancer cell proliferation and growth, facilitate cancer progression and metastasis by induce DNA replication stress, even genome instability. Activation of the cyclic GMP-AMP synthase (cGAS) mediates classical DNA sensing, is involved in genome instability, and is linked to various tumor development or therapy. However, the function of cGAS in gastric cancer remains elusive. In this study, the TCGA database and retrospective immunohistochemical analyses revealed substantially high cGAS expression in gastric cancer tissues and cell lines. By employing cGAS high-expression gastric cancer cell lines, including AGS and MKN45, ectopic silencing of cGAS caused a significant reduction in the proliferation of the cells, tumor growth, and mass in xenograft mice. Mechanistically, database analysis predicted a possible involvement of cGAS in the DNA damage response (DDR), further data through cells revealed protein interactions of the cGAS and MRE11-RAD50-NBN (MRN) complex, which activated cell cycle checkpoints, even increased genome instability in gastric cancer cells, thereby contributing to gastric cancer progression and sensitivity to treatment with DNA damaging agents. Furthermore, the upregulation of cGAS significantly exacerbated the prognosis of gastric cancer patients while improving radiotherapeutic outcomes. Therefore, we concluded that cGAS is involved in gastric cancer progression by fueling genome instability, implying that intervening in the cGAS pathway could be a practicable therapeutic approach for gastric cancer.

## Introduction

Gastric cancer (GC) is ranked among the top cancers worldwide due to its high incidence and mortality rate; nevertheless, China has the greatest GC occurrence and mortality [1]. It is well-known fact that the alteration in multiple signaling pathways fuels tumorigenesis. It confers unique characteristics on cancer cells, such as uninterrupted proliferation due to oncogenes activation, replication stress, and genome instability, as well as activation of the DNA damage response [2,3], and ongoing chronic DDR in cancer cells establishes selective pressure to maintain overgrowth of cancer cells, especially in the genetic or epigenetic defects.

Recently, cGAS has been identified as a critical DNA sensor [4], and cytosolic DNA triggers the cGAS–STING signaling axis, which catalyzes the formation of cGAMP [5], which is an important second messenger to activate the adaptor protein stimulator of interferon genes (STING) [6]. STING mediates the recruitment of TBK1, which subsequently activate the transcription factor IRF3 and induction of type I IFN response [7]. Thus, cGAS is critical in immune surveillance or tumor restraint [8,9]; Apart from acting as an anti-inflammatory and autoimmune regulator, cGAS also modulates autophagy [10] and senescence [11]; it also inhibits homologous recombination repair (HRR) of DSBs in a STING-independent manner [12,13]. Noteworthy, cGAS maintains the homeostasis of the aging cells and eliminates genetically altered and unstable cells during tumorigenesis. On the other hand, prolonged activation of the cGAS pathway promotes tumor formation by causing inflammation-driven tumorigenesis and metastasis [8,14]; nuclear cGAS also induces genome instability by inhibiting HRR to promote tumor development [12,15].

Considering the distinct functions of the cGAS in the regulation of GC development, we investigated the expression of cGAS using TCGA and CCLE databases and validated it in GC tissues and cell lines. Furthermore, we evaluated the function of cGAS by silencing its expression in GC cell lines; and clarified the underlying mechanisms of cGAS-mediated genome instability and tumor development.

## Materials and Methods

### Cell lines

The human immortalized gastric epithelial cell line GES-1, the human embryonic kidney epithelial cell line HEK293T, and the human GC cell lines AGS, MKN45, and NCI-N87 were purchased from the Shanghai Cell Bank of the Chinese Academy of Sciences (Shanghai, China), HEK293T and other cells were respectively cultured in DMEM or RPMI1640 (Gibco, Grand Island, NY, USA) medium supplemented with 10% FBS (Biological Industries, Beit Haemek, Israel), and cultured at 37°C in a humidified incubator provided with 5% CO_2_, and all cells were cultured in clean and mycoplasma or other infections free media.

### Patients

Human GC tumor and adjacent normal tissue specimens were collected from 41 patients who underwent partial gastrectomy or total gastrectomy in the Fifth Affiliated Hospital of Zhengzhou University. All the patients were diagnosed by following the WHO tumor classification & diagnostic criteria guidelines and the eighth edition of AJCC (American Joint Committee on Cancer, USA) GC staging. Retrospective clinical and pathological records of the 29 male and 12 female GC patients were also obtained. The ethical committee of Fifth Affiliated Hospital of Zhengzhou University has approved the sampling and experimental procedures and informed written consent was obtained from all patients. Moreover, we strictly followed the guidelines and ethical standards of the Helsinki deceleration of 1964 and its latest amendments.

### Data mining and analysis of database

The RNA-seqV2 data of 421 gastric tissues (containing 384 cancer and 37 normal tissues) and the complete clinical information were downloaded from The Cancer Genome Atlas (TCGA) database (https://cancergenome.nih.gov/); the differentially expressed genes (DEGs) screening and volcano plot were analyzed *via* chrislifescience (http://www.chrisapp.xyz:3838/R/AnnoE2/), using standard |log (fold change)| >1, adj P and a false discovery rate (FDR) < 0.05; the mRNA expression data of 37 GC cell lines were obtained from CCLE database (https://portals.broadinstitute.org). All mRNA expression values were log2-transformed and standardized. Genes co-expressed with cGAS were analyzed using the R (Pheatmap) and R (corrplot) software packages. The Kyoto encyclopedia of genes and genomes (KEGG) and gene ontology (GO) pathway enrichment analyses for the top 200 genes co-upregulated with *cGAS* in 384 GC patients were performed through DAVID Database for Annotation, Visualization and Integrated Discovery (https://david.ncifcrf.gov), and the significant biological functions of cGAS were studied. The prediction of cGAS’s interaction with other DDR proteins was performed by the GeneMANIA database (http://genemania.org/). Overall survival plot was generated using the Kaplan-Meier Plotter (http://kmplot.com/) based on the different levels of *cGAS* probe (1559051_s_at) in Affymetrix microarray gene expression data obtained from GSE15459, GSE51105and GSE22377; based on the median expression of *cGAS* in all GC tissues, the patients were divided into high and low-expression groups, and radiotherapeutic subgroups survival analysis was performed by using the Kaplan-Meier assay.

### Immunohistochemistry and H&E staining

We used our previously published protocol of immunohistochemistry [16]. Simply, the tissue sections were deparaffinized and hydrated in xylene, absolute ethanol, 95%, 85%, and 75% alcohol, and washed with PBS solution. For MKN45 cells, the tumor mass hematoxylin-eosin (H&E) staining was performed, and every section was immersed in hematoxylin and eosin staining solution. For the immunohistochemistry experiment, the antigen repairing and blocking was performed as described by the Mouse Streptavidin-Biotin Detection Kit (Zsbio, Beijing, China) manufacturer’s protocol, the cGAS antibody (Santa Cruz, Heidelberg, Germany) was incubated overnight at 4°C. On the next day, the primary antibody was washed in the PBS solution, biotinylated secondary antibody (Zsbio, Beijing, China) and horseradish-labelled streptavidin were added and incubated at 37°C; after diaminobenzidine staining and hematoxylin counterstained, all slides were placed in 75%, 85%, 95%, dehydrated in absolute ethanol respectively, and then sealed by neutral resin. The blank control group was incubated overnight with an equal amount of antibody dilution, and the other treatments were identical. According to the intensity of both the nuclei and membrane staining (blank = 0; light yellow = 1, yellow = 2, brown = 3) and the extent of stained cells (0% = 0, 1%–24% = 1, 25%–49% = 2, 50%–74% = 3, 75%–100% = 4) by the German semi-quantitative scoring system [17], each specimen was assigned a score, the immunoreactive score was the multiply of the intensity score with the extent of score of stained cells, ranging from 0 to 12. As to cGAS, we defined 0–3 score as low expression and 4–12 as high expression.

### RNA interference

Total 2 mL suspension containing 1 × 10^5^ cells in the logarithmic growth phase were seeded into 12-well plates and cultured for 24 h; at 80% confluence, the old medium was replaced with 0.8 mL serum-free RPMI1640 medium, and cells were transfected with siRNA using Lipofectamine-2000 (Thermo Fisher, Waltham, MA, USA). The complexes were prepared as follows: 2.5 µL Lipofectamine-2000 was diluted into 100 µL Opti-MEM I Reduced Serum Medium (Gibco), mixed gently, and incubated for 5 min at room temperature. Then 100 nM cGAS si-RNA or scrambled negative control (NC) (Santa Cruz) were added into 100 μL of Opti-MEM I Reduced Serum Medium separately and gently mixed. The diluted si-RNA and Lipo lipofectamine-2000 were mixed and incubated at room temperature for 20 min; subsequently, these complexes were added to each well-containing cell and medium. After transfection for 8 h, the mixture medium was replaced with a 1 mL complete medium, and after 48 h and cells were collected for cGAS knockdown efficiency by western blotting and other assays.

Lentiviral vectors with short hairpin RNA cGAS (sh-cGAS) and negative control (pLKO.1) were purchased from Youbio Tech (Changsha, China). The oligonucleotide encoding sequences were as follows: sh-cGAS-A:5’-GGAAGGAAATGGTTTCCAA-3’, and sh-cGAS-B: 5’-GCCTTCTTTCACGTATGTA-3’. For the stable silencing or overexpression of cGAS cell lines, HEK293T cells were seeded into a 6-well plate (4 × 10^5^ cells/well) and incubated for 24 h, transfected with 2 ug lentivirus plasmid sh-cGAS, Gag, and PMD2.G at 1:1:1 M/M/M ratio. Lentivirus suspension was collected after incubation for 48 and 72 h. Subsequently, AGS and MKN45 were infected with lentivirus and screened using 0.2 μg/mL puromycin after 48 h. After screening for 14 days, cells were analyzed for cGAS expression by western blotting and other analyses.

pcDNA3.1-HA–cGAS plasmid was obtained from Shanghai Key Laboratory of Tuberculosis [15], and GES-1 or AGS, MKN45 cells that stably expressed HA–cGAS were generated by transfection with pcDNA3.1-HA–cGAS plasmid and followed by 800 μg/mL and 200 μg/mL G418 (Thermo Fisher Scientific) selection.

### Cell proliferation

1 × 10^4^ cells were seeded into 96-well plates, and five replicate wells per assay, 10 μL of Cell Counting Kit-8 (Dojindo, Kyushu, Japan) were added into the well at cultured 0 h, 24, 48, 72 h, and the absorbance was measured at 450 nm by spectrophotometer after incubating at 37°C for 2 h in the dark.

For the 5-ethynyl-2′ -deoxyuridine (EdU) assay, 5 × 10^4^ cells were cultured on the coverslips in 24-well plates. After culturing for 24 h cells, the cells were stained with EdU Cell Proliferation Assay Kit (Sangon Biotech, Shanghai, China) by following the manufacturer’s instructions. Simply, the cells were replaced complete medium containing 50 μM EdU and incubated for 2 h, then fixed with 4% paraformaldehyde and permeabilized with 0.5% Triton X-100, and incubated with EdU fluorescence staining solution for 30 min. The cell nuclei were stained by hoechst33342 solution (Solarbio, Beijing, China). The cells stained with EdU were visualized and photographed by using the Airscan microscope (Carl Zeiss880, Oberkochen, Germany). EdU positive foci were counted and compared with control.

### Cell migration and wound healing assay

Cell migration and wound healing assay were performed to identify the effect of si-cGAS on cell migration. Briefly, 2 × 10^5^ cells were suspended in 200 μL RPMI1640 without serum and seeded into the upper part of the 8μm pore transwell (Corning, New York, NY, USA); meanwhile, 600 μL RPMI1640 with 10% FBS was supplied into the lower chamber as a chemoattractant. After culturing for 48 h at 37°C, the upper part of the transwell membrane was washed thrice with PBS and gently wiped with a cotton swab, and the membrane was immersed into 4% paraformaldehyde for 30 min, and stained with 0.1% crystal violet for 20 min. Then the upper part of the transwell membrane was wiped off with a cotton swab. Finally, cells attached to the membrane were stained, photographed by inverted microscopy (Carl Zeiss), and quantified. In wound healing assay, ~2 × 10^5^ cells/well were seeded into 12-well plates, after culturing for 12 h and when cells reached the 90% confluence, the linear scratch wounds were made as a monolayer in each well by using 10 μL sterile pipette tip. Cells were washed thrice with 1 mL PBS to remove the solitary cell, and photographed, the cultured for the next 48 h in serum-free RPMI1640 medium, then changes in the wound’s width were observed and photographed with inverted microscopy and measured by Image J software; the wound healing rate was calculated with the following equation:

Wound healing rate = (W_0h_ − W_48h_)/W_0h_ × 100%

W_0h_ and W_48h_ are the wound width at 0 and 48 h, respectively.

### Cell apoptosis

Flow cytometry was performed to analyze cell apoptotic cells in the cGAS knockdown cells population. The apoptosis rate was determined using the Annexin V-FITC apoptosis analysis kit (Sungene Biotech, Tianjin, China). Briefly, after cells were transfected by cGAS siRNA at 100 nM for 72 h, cells were harvested with EDTA null trypsin and centrifuged at 100 g for 5 min, washed thrice with PBS. Subsequently, cells were stained with Annexin V-FITC and PI for 5 min. Finally, the flow cytometer BD Accuri C6 (BD Biosciences, Franklin Lakes, NJ, USA) and Flowjo10 software were used to analyze data that determined the apoptosis rate.

### Xenograft

Female BALB/c-nu nude mice were purchased from the China Charles-river Company (Beijing, China). For the xenograft assay *in vivo*, 5 × 10^6^ MKN45-sh-cGAS and sh-control cells were subcutaneously injected into the right dorsal flank of five-week-old male BALB/c nude mice. Tumor sizes were measured every other day, and the volume was calculated by (length × width^2^)/2. After 14 days of cells’ injection, all mice were anesthetized with 3% isoflurane and euthanized by cervical dislocation, and the tumors were harvested and photographed.

### Comet assay

After 48 h of transfecting GES-1 cells with pcDNA3.1 or HA-cGAS, 1 × 10^5^ cells/mL were collected and resuspended in Dulbecco’s phosphate-buffered saline (DPBS, Gibco). Cells were treated with 50 μM of genotoxic agent etoposide for 2 h, then comet assay was performed by following the manufacturer’s instructions (Trevigen, Gaithersburg, MD, USA). DNA damage was assessed by measured tail moments using comet-score software (casplab_1.2.3b2).

### Western blotting

Cells were subjected to lysis through protein lysis buffer containing 150 μL of Radio Immunoprecipitation Assay lysate buffer (Gibco), 1% PIC inhibitor (Gibco), and SDS loading buffer (Solarbio). Protein samples were electrophoresed through SDS-PAGE gel. After electrophoresis, the samples were transferred to the Polyvinylidene fluoride (PVDF) membrane, followed by blocking with 5% skimmed milk prepared in TBST for 1 h. After that, membranes were washed with TBST thrice for 5 min each, then incubated overnight at a 4°C shaker with primary antibodies prepared in 5% bovine serum albumin (BSA, Becton, Dickinson, and Company, Franklin Lakes, NJ, USA). The next day, primary antibodies were removed and washed thrice with TBST followed by incubating with respective secondary antibodies prepared in TBST containing 5% skimmed milk for 1h at room temperature. Membranes were washed, ECL (Thermo Fisher Scientific) mixture was added to membranes, and images were visualized, adjusted, and captured by Bio-Rad ChemiDoc™ XRS+ System (Bio-Rad, Hercules, CA, USA).

### Co-Immunoprecipitation (Co-IP)

The MRN complex and cGAS co-IP assays were carried out according to the manufacturer’s instructions (Classic Magnetic Protein A/G IP/Co-IP Kit; Epizyme, Shanghai, China). To summarize, specific antibodies (anti-cGAS, Santa Cruz, dilution:1:50 and anti-NBN, Proteintech, Rosemont, IL, USA, dilution: 4 µg/mL) were mixed with protein A/G Magnetic Beads and placed on the rotator overnight at 4°C to generate antibody-magnetic bead complexes; IgG was used as a negative control. Proteins were extracted from AGS cells in 60 mm dishes and lysed using mild lysis for 30 min, then incubated with antibody-magnetic beads complexes overnight at 4°C to form protein-antibody-magnetic beads complexes; the immunoprecipitation complexes were washed and used for further immunoblotting (IB) analysis.

### Reverse transcription-quantitative PCR (RT-qPCR)

Cells were transfected by cGAS siRNA or NC at 100 nM; After 48 h of siRNA treatment, cells were digested in TRIzol reagent (Invitrogen, Waltham, MA, USA). 1 µg total RNA was converted into cDNA using ReverTra Ace qPCR RT Kit (Toyobo, Osaka, Japan) according to the manufacturer’s instruction. Roche Lightcycler480II system (Roche, Basel, Switzerland) was employed to perform qPCR with 2 µL of cDNA and QuantiNova SYBR Green qPCR Mastermix (Qiagen, Dusseldorf, Germany) based on the manufacturer recommendations. Primers sequences were designed in NCBI Primer-BLAST, GAPDH was used as a reference gene. The relative expression of the transcripts of cGAS was determined using the 2^−ΔΔCt^ method.

### Immunofluorescence (IF)

To rule out the effect of mitosis on the intracellular localization of cGAS, cells were transfected with the HA-cGAS and seeded into a 24-well plate (5 × 10^4^ cells/well) with pre-placed coverslips and cultured for 24 h. At 60% confluence, cells were replaced with serum-free medium, and exposed to X-Ray (dose of 8 Gy); after 3 h of IR treatment, cells were washed and fixed in 0.5 ml 2% paraformaldehyde, blocked with 250 μL blocking buffer and incubated with 150 μL of primary antibody in blocking buffer for 1 h at room temperature followed by washing and 1 h room temperature incubation with secondary antibody (150 μL) prepared in blocking buffer. Finally, to stain the nucleus a drop of DAPI-containing mounting media was poured on the slide and dispersed across the entire slide by tilting it upside down. The fluorescence of cGAS was observed and imaged under a Confocal Laser Scanning Microscope (Carl Zeiss880).

### Statistics

All statistical analyses were performed using SPSS 25.0 software (SPSS, Chicago, IL, USA). Two-tailed Student’s *t*-test was used to analyze the differences in gene expression value of the TCGA database between the groups; For cell function assay, data were determined by a two-tailed Student’s *t*-test or Wilcoxon Signed Ranks; the IHC data were evaluated by the Wilcoxon matched-pairs signed-ranks sum test, all function experiments were performed in triplicate. The correlation between the cGAS and pathological conditions of the GC patients was analyzed using the χ^2^ or Yate’s correction χ^2^ test. The GEO2R online-analysis menu was used to analyze the data from the GEO database. Survival analysis was performed by using the log-rank test. *P*-value < 0.05 was considered the significant.

## Results

### The upregulation of cGAS in GC tissues and cell lines

**Figure 1:**
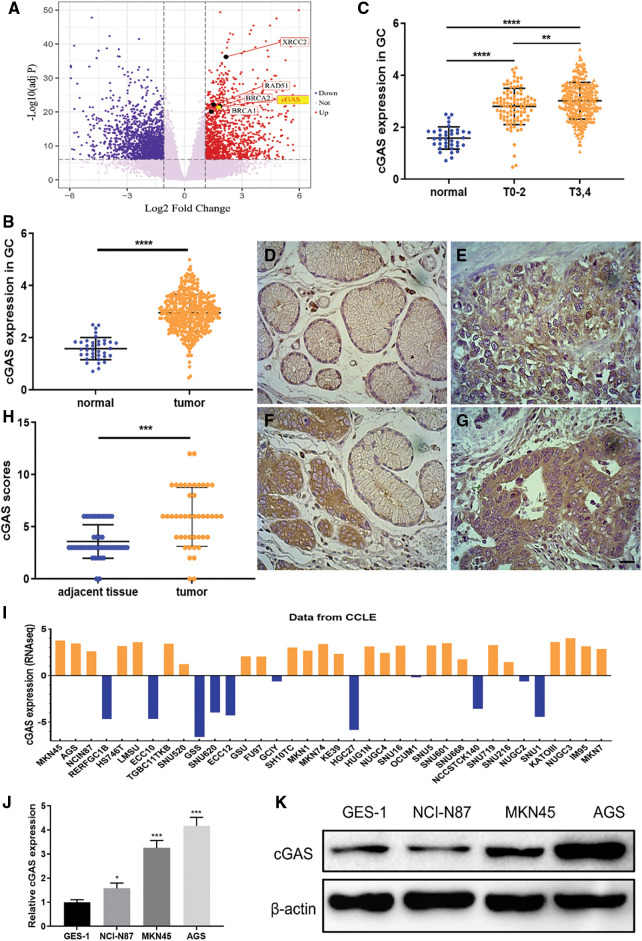
High expression of cGAS in gastric cancer tissues and gastric cancer cell lines. **A**. Volcano map of gastric cancer and normal tissue showing differentially expressed genes in TCGA database (cGAS and several vital genes of HR repair were specially labelled). **B**. The expression data of cGAS in gastric cancer tissues and normal human gastric mucosa in the TCGA database. **C**. The level of cGAS expression in different T stages of gastric cancer. **D–G**. Representative immunohistochemical results: D. adjacent tissue, score 2 (scale bar:20 μm); **E**. Cancer tissue, score 4; F. Cancer tissue, score 8; **G**. Cancer tissue, score 12. **H**. Retrospective immunohistochemical analysis score of cGAS in gastric cancer tissues and adjacent tissues. **I**. cGAS expression data for 37 gastric cancer cell lines in the CCLE database. **J**. RT-qPCR for cGAS relative expression of mRNA in 3 gastric cancer cell lines and GES-1 cell line (n = 3). **K**. Western blotting of cGAS in three gastric cancer cell lines and GES-1 cell line (* *p* < 0.05, ** *p* < 0.01, *** *p* < 0.001, **** *p* < 0.0001).

To assess the status of cGAS in GC, we compared the expression level of cGAS in GC tissues with normal gastric mucosa using RNA-seq data obtained from the TCGA databases. Setting a screening threshold, we identified a total of 3443 differentially expressed genes (DEGs), with 1611 genes upregulated and 1832 genes downregulated (Fig. 1A). The expression of the cGAS gene in 384 GC samples and 37 normal gastric mucosae was shown to be considerably greater in GC (Fig. 1B), notably in T3&4 stage tumors (Fig. 1C). Retrospective immunohistochemical data of 41 GC and surrounding tissues demonstrated a greater expression of cGAS in GC tissue, which was used to further validate cGAS expression (Figs. 1D–1H). With an 85.3% positive rate, cGAS was broadly disseminated in the nucleus and cytoplasm of tumor cells (Table 1). Furthermore, the CCLE database’s RNA-seq data revealed that cGAS was substantially expressed in 26 of 37 GC cell lines (Fig. 1I). At both the mRNA and protein levels, RT-qPCR and WB tests demonstrated that the expression of cGAS in the three GC cell lines was higher than that of the “typical” gastric epithelial cell line GES-1 (Figs. 1J and 1K). These findings imply that cGAS increase and aberrant activation are likely to contribute to the development of GC.

**Table 1:** The frequency of cGAS in gastric cancer tissue and adjacent tissue

Classification	Total	cGAS (+)	cGAS (−)	*P* value
Tumor tissue	41	35(85.3%)	6(14.7%)	<0.001
Adjacent tissue	41	13(31.7%)	28(68.3%)	

### Knockdown of cGAS inhibits tumor cell growth in vitro *and* Vivo

**Figure 2:**
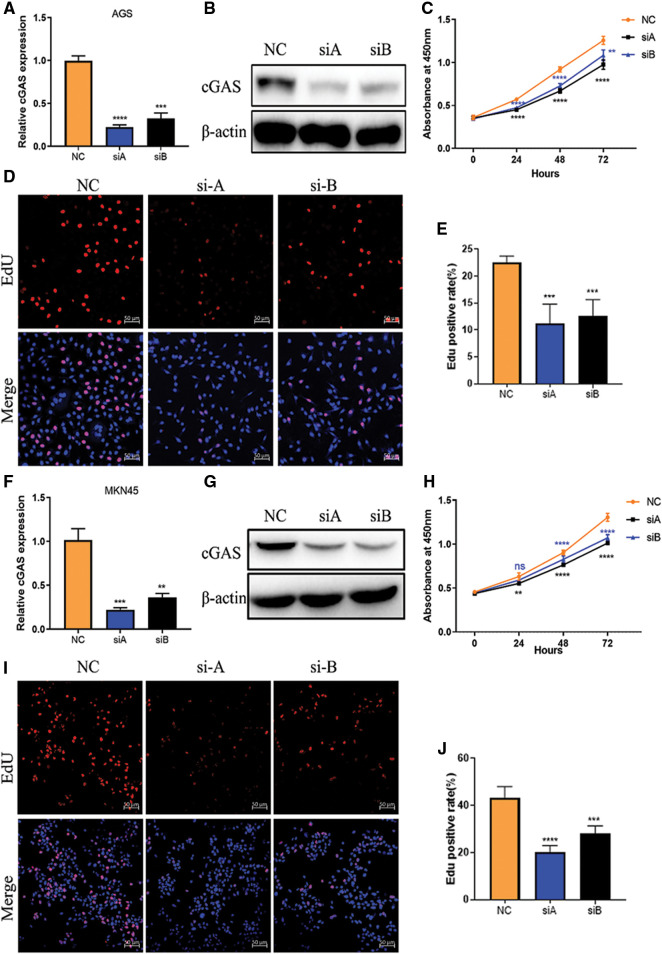
cGAS knockdown inhibits GC cells proliferation *in vitro*. **A**, **B**. The relative mRNA expression and protein expression of cGAS after siRNA triggered knockdown in AGS cells. **C**. CCK-8 assay show reduced cell proliferation in si-cGAS AGS cells. **D**, **E**. EdU assay of AGS cells in vitro, EdU (red) and hoechst33342 (blue). **F**, **G**. The cGAS knockdown efficiency in MKN45 cells. **H**. cell proliferation curve of si-cGAS MKN45 cells. **I**, **J**. EdU assay of MKN45 cells *in vitro* (**p < 0.01, ***p < 0.001, ****p < 0.0001, n = 3).

To investigate whether cGAS exert functional roles in GC cells, we chose the AGS cell line which showed higher expression of cGAS and knockdown of cGAS by using siRNA. RT-qPCR and WB assays demonstrated that siRNA significantly reduced transcription as well as translation of cGAS (Figs. 2A and 2B). CCK-8 assay showed that knockdown of cGAS markedly inhibited the cell viability of AGS cells (Fig. 2C); EdU incorporation assay demonstrated that knockdown of cGAS resulted in the reduced DNA replication in AGS cells (Figs. 2D and 2E).

**Figure 3:**
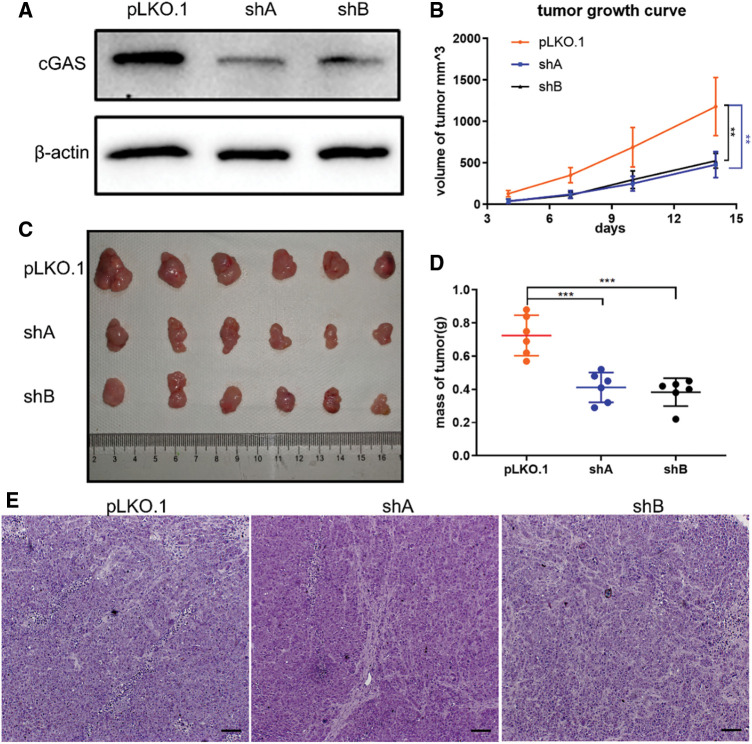
cGAS knockdown inhibits the proliferation of MKN45 cell line *in vivo*. **A**. The cGAS knockdown efficiency in MKN45 cells. **B**. Subcutaneous tumor volume, the growth curve of sh-cGAS MKN45 cells in nude mice. **C**. Representative images showing the tumors’ sizes were dissected 14 days after subcutaneous injection of sh-cGAS MKN45 and control cells. **D**. Subcutaneous tumor mass quality. **E**. H&E staining of MKN45 tumor tissues (**p < 0.01, ***p < 0.001, scale bar:100 μm).

To validate the above data, we performed the same experiments on another gastric cancer cell line MKN45 showing upregulation of cGAS, encouragingly, MKN45 cells with cGAS knockdown exhibited consistent cell viability and slowed DNA replication (Figs. 2F–2J). We further constructed a stable cGAS-knockdown MKN45 cell line (Fig. 3A). Furthermore, knockdown of cGAS significantly decreased tumor mass and growth rate in BALB/c-nude mice (Figs. 3B–3D), meanwhile, H&E staining of tissues confirmed the tumors were induced by the injected GC cells (Fig. 3E). Taken together, cGAS promoted tumor growth by regulating DNA replication **in vivo* and vitro*.

**Figure 4:**
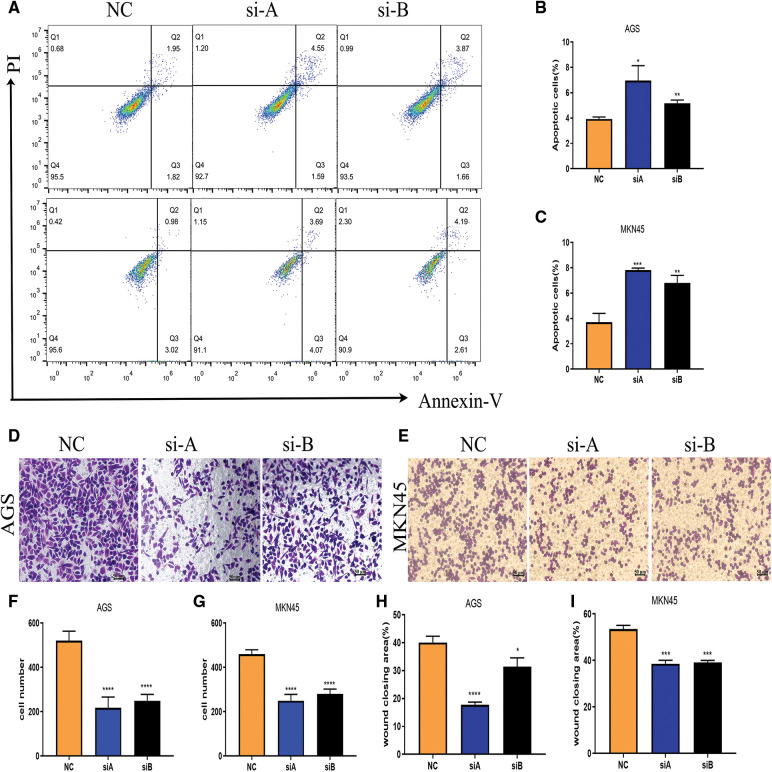

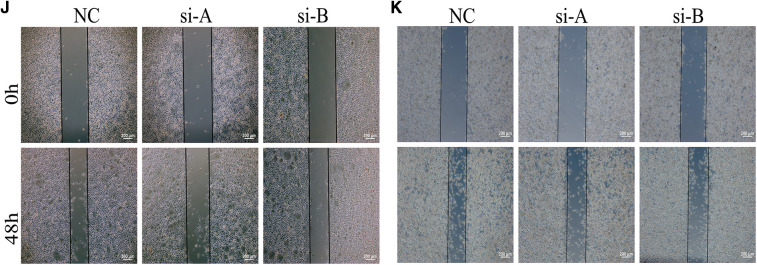
Knockdown of cGAS affects gastric cell function. A–C. Flow cytometry assay detected moderate apoptosis in si-cGAS AGS (up) and MKN45(down) cells. D, E. si-cGAS reduced the migration of AGS (left) and MKN45(right) cells. F, G. Several migrated AGS and MKN45 cells. H–K. Wound healing observed slow migration of si-cGAS AGS (left) and MKN45(right) cells. (*p < 0.05, **p < 0.01, ***p < 0.001, ****p < 0.0001, n = 3).

Of note, Flow Cytometry analysis for apoptosis indicated that cGAS knockdown enhanced the apoptosis of GC cells (Figs. 4A–4C). Moreover, the Transwell assay demonstrated that the cell migration rate of the si-cGAS groups was significantly lower than that of the NC group in both AGS and MKN45 cells (Figs. 4D–4G). Consistently, knockdown of cGAS marginally reduced the speed and quality of wound healing in GC cells (Figs. 4H–4K). The above data indicated that cGAS is involved in regulating cell migration and apoptosis of GC cells.

### cGAS regulating MRN complex formation and cell cycle checkpoint

**Figure 5:**
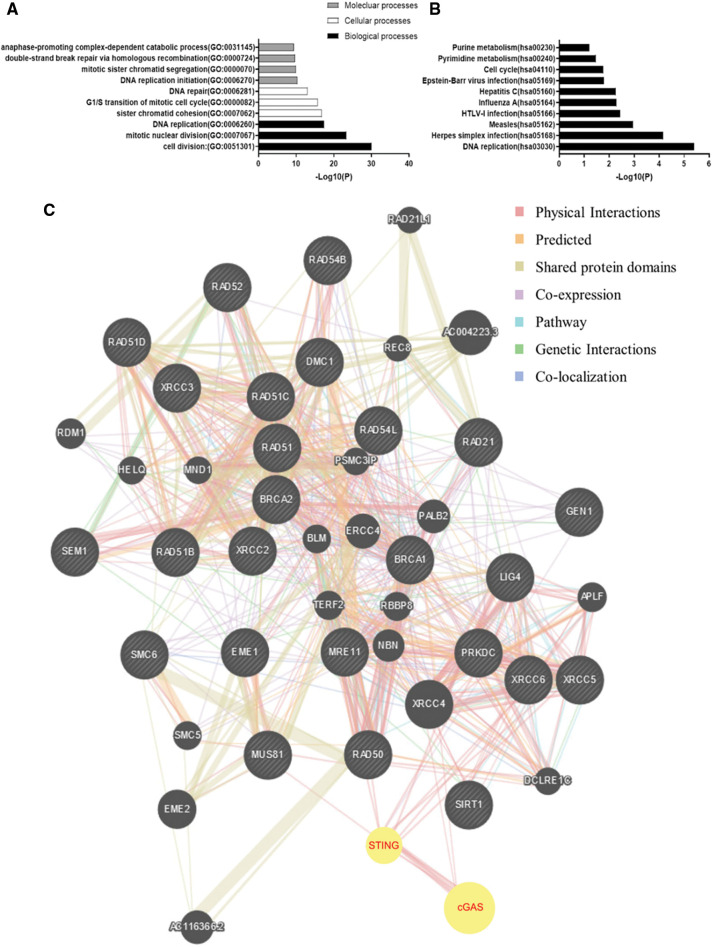
Bioinformatics analysis reveals the role of cGAS in DNA damage repair. **A**, **B**. GO and KEGG functional analysis of the top 200 co-expressed genes with cGAS from the TCGA dataset. **C**. Functional proteins’ interaction networks display potential physical interaction between cGAS and MRN complex.

**Figure 6:**
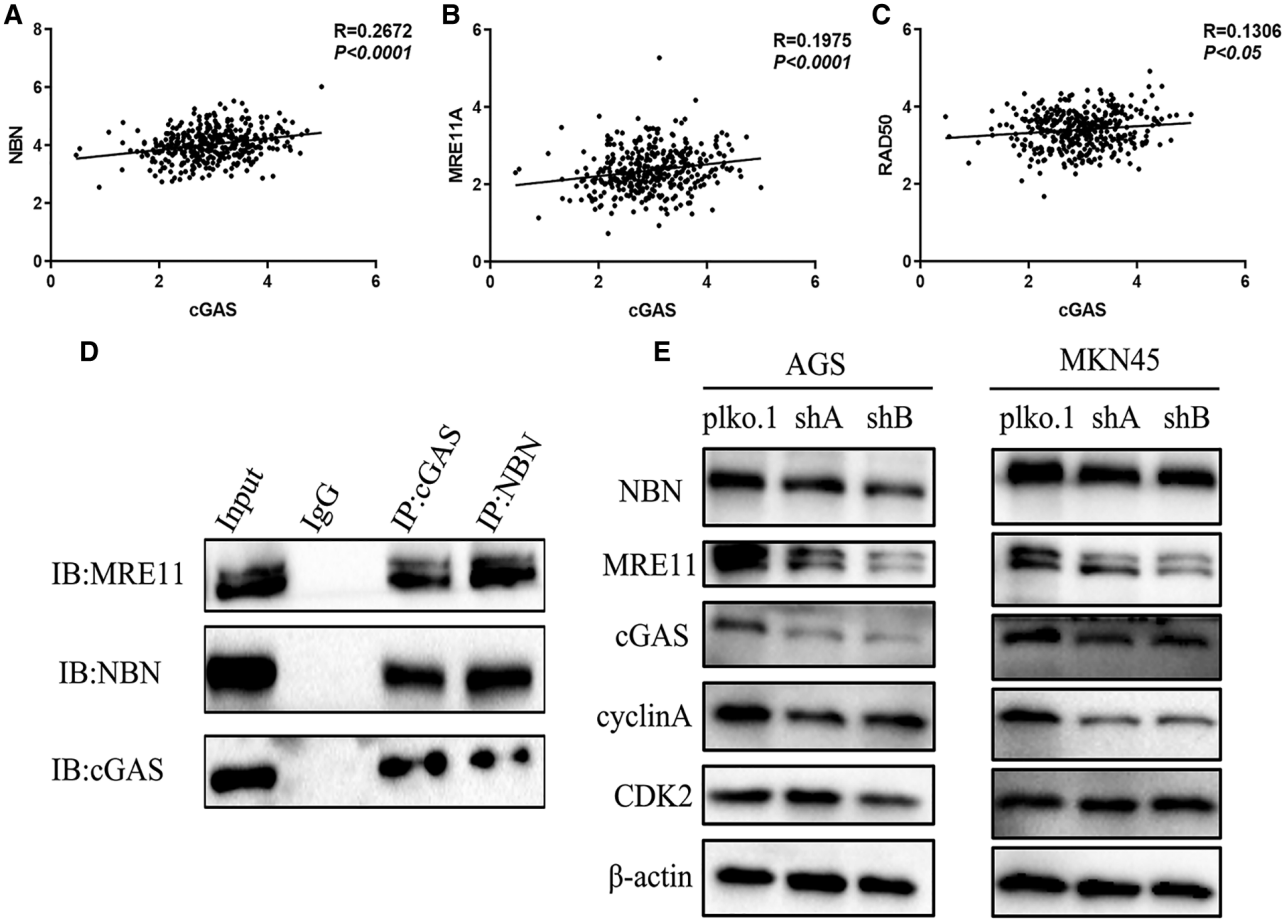
cGAS were regulating MRN complex formation and cell cycle checkpoint. **A–C**. The correlation of the MRN complex subunit expression with cGAS. **D**. Co-IP experiment and follows immunoblotting (IB) assay revealed that cGAS interacted with NBN and MRE11 in AGS cells. **E**. Western blot results of the MRN complex and cell cycle checkpoint proteins in cGAS knockdown AGS and MKN45 cells.

To interrogate the mechanism by which cGAS affects GC cellular function, we performed pathway enrichment analysis. The GO analysis of top cGAS-related signatures revealed their role in double-strand break repair *via* homologous recombination, DNA replication, and G1/S transition of the mitotic cell cycle. However, cell division and DNA replication were observed as the most enriched biological functions; these functions were consistent with the results in the molecular process: DNA replication and DNA repair (Fig. 5A). Furthermore, the KEGG enrichment analyses revealed their role in DNA replication, Herpes simplex infection, and HTLV-I infection (Fig. 5B). Based on these findings, we constructed an associated network between these genes’ encoded proteins and found that cGAS-STING physically interacted with RAD50, MRE11, and NBN (Fig. 5C); these three proteins activate and form the MRN complexes in response to the initial and sustained DSBs, stalled replication forks, and help orchestrate cell cycle progression and damage response [18]. Interaction of MRE11 and NBN has been demonstrated [18], and the co-expression data in GC tissues showed high co-expression between cGAS and NBN or MRE11 (Figs. 6A–6C). Endogenous proteins that bind to cGAS were immunoprecipitated from AGS cell lysates by cGAS antibody, and the expression of MRE11 and NBN in the isolated proteins was detected by immunoblotting. We also detected MRE11 and cGAS in the lysates when immunoprecipitated by NBN antibody, implying that cGAS may be involved in the formation of the MRN complex (Fig. 6D). We identified the expression of MRN complex proteins and found consistent downregulation of MRE11 and NBN in cGAS-knockdown cells (Fig. 6E), further inhibiting the expression of its downstream effectors, cycle checkpoint-associated proteins cyclin-A instead of CDK2, thereby inhibiting intracellular G1/S phase transition and DNA synthesis, which may be responsible for the reduction in DNA replication (Fig. 6E).

### cGAS is involved in DNA HR repair and genomic instability

**Figure 7:**
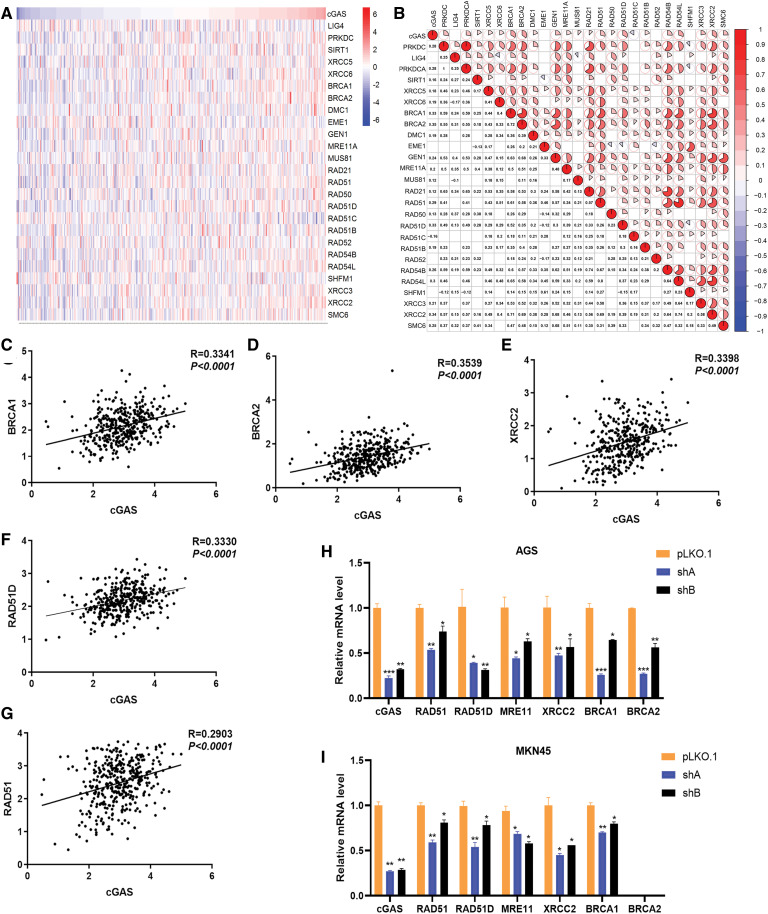

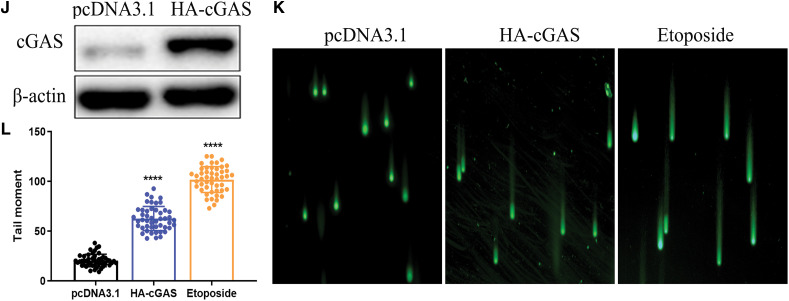
cGAS is involved in DNA HR repair and promote genomic instability. A. Heat maps of cGAS and HR repair genes’ expression; B. The Pearson correlation between cGAS and HR repair genes in the TCGA database represents the correlation coefficient value. C–G. Five HR repair genes with the highest correlation with cGAS in gastric cancer. H, I. RT-qPCR results of HR repair genes co-expressed with cGAS in sh-cGAS AGS and MKN45 cells. J. Western blot results of cGAS overexpression. K. Comet assay determined the genome stability in GES-1 cells with cGAS overexpression. L. Comet tail moment length. (*p < 0.05, **p < 0.01, ***p < 0.001, ****p < 0.0001)

Based on the bioinformatics analysis, we hypothesized that cGAS might be involved in regulating DNA damage response. Therefore, we conducted a co-clustering analysis of gene expression data in tumor tissues of 384 GC patients in the TCGA database. Heat-map analysis revealed a significant correlation between the expression of cGAS with DNA HR repair-related genes in GC tissues (Fig. 7A and 7B). The top related five genes were BRCA1, BRCA2, XRCC2, RAD51, and RAD51D (Figs. 7C–7G), thus approving our hypothesis. In addition, the activation of DDR by cytoplasmic cGAS induced expression of DNA repair proteins, and we further validated the expression of these genes in cGAS knockdown AGS and MKN45 cells by RT-qPCR; interestingly, downregulation of these genes in cGAS knockdown cells was observed, except for the deficient of BRCA2 in MKN45 cells (Figs. 7H and 7I). To further validate the effect of cGAS on genome instability in GC cells, we overexpressed cGAS in the GES-1 cell line (GES-1-HA-cGAS) (Fig. 7J), which showed a longer comet tail (Figs. 7K and 7L), indicating increased DSBs. These data demonstrate that cGAS is involved in DNA HRR in GC.

### cGAS and prognosis of GC patients

**Figure 8:**
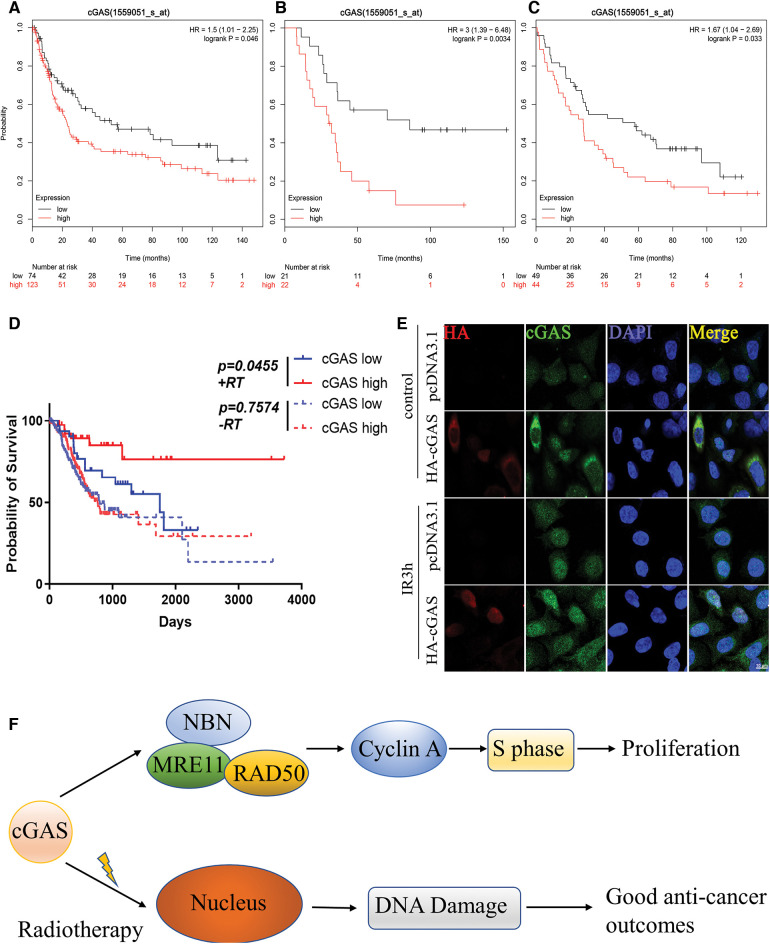
cGAS cause poor prognosis in GC patients. **A–C**. K-M survival curve demonstrating the overall survival times of GC patients with different cGAS expressions. The datasets were derived from GSE15459, GSE51105, and GSE22377 in the GEO database, respectively (scale bar: 10 μm). **D**. K-M survival curve presenting the overall survival times of GC patients from the TCGA database stratified based on the treatment (radiotherapy (RT) and non-radiotherapy) and expression levels of cGAS (cutoff: median value). **E**. Immunofluorescence of endogenously and exogenously localization of cGAS after IR exposure (scale bar: 10 μm). **F**. The schematic diagram of cGAS in gastric cancer progression and radiotherapy.

We analyzed the relationship between the expression of cGAS and the prognosis of GC patients in the K-M plotter database. The K-M survival analysis of 1559051-s-at chip data of GSE15459 (*p* = 0.046), GSE51105 (*p* = 0.033), and GSE22377 (*p* = 0.0034) found a low long-term survival rate in GC patients with high expression of cGAS (Figs. 8A–8C). Further grouping indicated that high cGAS expressing GC patients treated with radiation displayed prolonged survival; however, in patients who never received radiation treatment, the expression levels of cGAS did not affect the survival (Fig. 8D). We also detected nuclear translocation of endogenous or exogenous cGAS in AGS cells following IR exposure, confirming that cGAS was dispersed in both the nucleus and cytoplasm of cells (Fig. 8E). The increase in nuclear translocation could be a key node in cGAS’s function. We recently reported that cGAS suppresses HR repair in the nucleus [15], therefore we hypothesize that cGAS nuclear translocation enhances DNA damage in GC tumor cells, which could explain their increased radiation sensitivity. We finally analyzed the correlation between cGAS and the clinicopathological characteristics using TCGA data and in-house IHC data. The data demonstrated that the association of high cGAS expression with invasion depth of the tumors (*p* = 0.032) but not related to gender (*p* = 0.409), age (*p* = 0.279), Lauren Classification (*p* = 0.385), lymph node metastasis (*p* = 0.323), tumor metastasis (*p* = 0.789) and tumor grade (*p* = 0.761) of TCGA data (Table 2). Unfortunately, we did not find any significant difference in gender (*p* = 0.691), age (*p* = 0.523), degree of differentiation (*p* = 0.123), depth of invasion (*p* = 0.648), or lymph node metastasis (*p* = 0.383), and tumor grade (*p* = 0.849) (Table 3). Therefore, we uncovered the dual function of cGAS in development of GC and radiotherapy sensitivity (Fig. 8F).

**Table 2:** Correlation between cGAS expression and clinicopathological features of gastric cancer in TCGA datasets

Characteristics	n	cGAS relative expression		Statistics	*P* value
		low(n)	high(n)		
**Gender**					
male	251	121	130	0.681	0.409
female	133	70	63		
**Age(years)**					
<65	178	94	84	1.173	0.279
≧65	197	93	104		
**Lauren classification**					
stomach	215	103	112	0.755	0.385
intestinal	168	88	80		
**T stage**					
0,1,2	98	58	40	4.579	**0.032**
3,4	283	132	151		
**LN meta**					
with	119	63	56	0.979	0.323
without	255	184	134		
**M stage**					
0	345	170	175	0.072	0.789
1	20	11	9		
**Stage**					
I, II	172	87	85	0.093	0.761
III, IV	200	98	102		

**Table 3:** Correlation between cGAS expression and clinicopathological features of gastric cancer

Characteristics	n	cGAS relative expression		Statistics	*P* value
		low(n)	high(n)		
**Gender**					
male	28	9	19	0.158	0.691
female	13	5	8		
**Age(years)**					
<65	18	6	12	0.009	0.523
≧65	23	8	15		
**Differentiation**					
Poor-Undiff	24	11	13	2.374	0.123
Well-Mid	17	3	14		
**T stage**					
0,1,2	9	2	7	0.208	0.648
3,4	32	12	20		
**LN meta**					
with	17	4	13	0.761	0.383
without	24	10	14		
**Stage**					
I, II	11	3	8	0.036	0.849
III, IV	30	11	19		

## Discussion

Gastric cancer ranked fifth in terms of incidence and fourth in cancer-related death worldwide. It causes global severe economic and public health burdens, while its’ pathogenesis remains elusive [1]. In the last decades, genome instability has been recognized as a crucial mechanism of gastric carcinogenesis [19,20]. Oncogenes drive cancer cell proliferation to induce DNA replication stress, generate DSBs and activate DDR which induce genome instability and selective escape from the apoptosis [21], thereby promoting tumor progression. GC has recently been identified as a subgroup of the tumors that exhibit defects in HR repair pathways [22], showing high rates of genome instability and poor prognosis [23]. Therefore, therapies targeting genome instability have high prospects of applications in GC treatment.

As an intracellular DNA sensor, cGAS detects or mislocalized double-stranded DNA without sequence specificity [24]. Hence, the activation of cGAS triggers various physiological processes. In the cytoplasm, it dominantly promotes the 2’3-cGAMP production and subsequently activates STING, then triggers inflammation and antitumor immunity [9,25], senescence [26] or autophagy [10], so acts as a tumor suppressor [27]. However, chronic stimulation of the cGAS-STING pathway may induce inflammation-driven tumorigenesis [14]. Recent studies have also reported that cGAS accumulates in the nucleus during mitosis [11] and genomic stressed [15]; nuclear cGAS interact with the histone 2A/2B and is tightly anchored to the “acidic patch” to prevent autoreactivity to self-DNA [28], which causes limited responses to endogenous DNA. The exclusive behavior of cGAS highlights that nuclear cGAS may participate in chromatin architecture modulation. Specifically, the nuclear-localization of cGAS has essential significance in cancer progression and therapy, tumors with high tolerance to DNA damage may provide a permissive microenvironment for nuclear cGAS to respond to the genotoxic stress to exert its tumorigenic effects [29]. As we previously reported, nucleic cGAS interferes with HRR by inhibiting the Timeless/PARP1 complex and promotes the proliferation of LLC cells [15]; Also, cGAS may attenuate HRR by interfering with RAD51-mediated formation of displacement loop (D-loop) and DNA strand invasion in HEK293T cells [12]. A positive correlation was reported between nuclear cGAS and micronucleus generation in mice [12] and GI in HCA2-H15c cells [15]. These data revealed a dual function of cGAS in different tumorigenesis [9,14,15,27,30]. It may be a key factor for different biological functions, such as subcellular localization of cGAS [31]. However, the role of cGAS in GC has not been reported.

In the current study, we discovered the high expression of cGAS in GC tissues and 26 GC cell lines, suggesting inactivation of the cGAS in GC due to the high chromosomal instability (CIN), which produces a large amount of cytoplasmic dsDNA in cancer cells [32]. Therefore, we hypothesized that cGAS might be a protooncogene in GC. Additionally, we chose three gastric cancer cell lines: AGS, MKN45, and NCI-N87, interestingly cGAS showed higher mRNA levels than normal GES-1 cells, while NCI-N87 had nearly the same protein expression as GES-1. AGS and MKN45 are low/moderately differentiated tumor-derived cell lines, whereas NCI-N87 is highly differentiated cells [33], it may have some unknown post-translational and post-translational modifications that result in different mRNA and protein levels. As a result, in the follow-up study, we chose AGS and MKN45 cells. As assumed, the silencing of cGAS led to decreased cells proliferation, migration, and increased apoptosis in AGS and MKN45 cells. Previously, it has been reported that silencing cGAS decrease the viability of BT-549 cells [34]; furthermore, our xenograft experiment in BALB/nu mice also demonstrated a decrease in tumor growth in sh-cGAS MKN45 cells. To explore how cGAS exert the proliferation of GC cells, we performed GO and KEGG functional enrichment analysis, which showed its involvement in DNA replication and repair. Associated protein network displayed potential physical interaction between cGAS-STING and MRN complex subunit: RAD50, MRE11, and NBN; Gene co-expression analysis also revealed high co-expression of cGAS and these three in GC tissues. The MRN complex is one of the initial sensor and responder of the DNA damage and orchestrates the DDR [18], which recognize the DSB terminus and trigger the cell cycle checkpoint response, recruits DNA repair genes in response to DNA damage [35] by interacting with the protein kinase Ataxia Telangiectasia-Mutated (ATM). The CoIP results demonstrated that cGAS could also interact with MRE11/NBN in AGS cells; We found that the cGAS knockdown was accompanied by down-regulation of NBN and MRE11, and cell cycle arrest was observed at the G1 phase due to downregulation of cyclin A, thus explaining the role of cGAS in the regulation of cellular function and DNA replication in GC cells. These data suggest an exciting function of cGAS: the aberrant activation of cGAS in GC cells maintains their damaged DNA replication and cell cycle progression. In addition, cGAS recognizes cytoplasm dsDNA, an early event in DDR activation in GC cells [27], follows the activation of DSB repair and recruits repair factors such as RAD51; cGAS deletion impairs the initiation of DDR and prevents the activation of downstream DNA repair factors such as *RAD51, XRCC2, BRCA1*, etc. the interplay of cGAS with chromatin may be the critical mechanism [36] and warrants further investigation. A proteomics study also suggested that cGAS is associated with DNA-PK, the other molecular sensor for DNA damage [37], which highlights the possible role of nuclear cGAS in modulation of chromatin architecture [29].

The comet assay results showed that the GES-1 cells with overexpressing cGAS exhibited a higher level of GI, suggesting that cGAS is involved in regulating genome stability in GC cells. Therefore, we studied the impact of cGAS expression on the prognosis of GC patients. K-M survival analysis of the GEO database showed that GC patients with high cGAS expression had a worse prognosis [38], and nuclear accumulation of MRN complexes is associated with gastric cancer progression and poor prognosis [39], which is consistent with our results. However, it was found that patients with high expression of cGAS had a better response to radiotherapy, which suggests a dual function of cGAS on tumor progression and treatment response. It has been reported that nuclear enrichment of cGAS induced by IR and DNA damage agents inhibits HRR [15], allowing further accumulation of DNA damage in cancer and establishing a feed-forward loop of chromosomal instability [40], IR-induced nuclear displacement of exogenous and endogenous cGAS was also observed in the GC cells, which highlights an exciting role of nuclear cGAS in promoting tumors sensitivity to the DNA damaging agents. These data confirmed our perspective and were consistent with the results in ovarian cancer and non-small cell lung cancer [41], suggesting that patients with high expression of cGAS have better radiosensitivity, which may provide an additional significance for the clinical application of cGAS in GC therapy.

In conclusion, our findings revealed that abnormally high expression of cGAS in GC tissues and cell lines may promote cellular DNA damage and genome stability, ultimately leading to GC development. This process may be associated with the inhibition of HRR through nuclear cGAS. According to different subgroups’ survival curve results, cGAS expression may serve as an independent prognostic marker for GC patients and a predictor of radiotherapeutic effectiveness. Our study enriches the functional importance of cGAS in GC; however, given the functional intricacy of cGAS in the cytoplasm and nucleus, the adoption of cGAS as a potential therapeutic target required more detailed and rigorous studies.

## Data Availability

The datasets used and analyzed during the current study are available from the TCGA and GEO database.
